# *Geranium thunbergii* extract-induced G1 phase cell cycle arrest and apoptosis in gastric cancer cells

**DOI:** 10.1080/19768354.2019.1699161

**Published:** 2019-12-04

**Authors:** Hana Lee, Woong Kim, Hyeon-Gu Kang, Won-Jin Kim, Seok Cheol Lee, Seok-Jun Kim

**Affiliations:** Department of Biomedical Science & BK21-Plus Research Team for Bioactive Control Technology, Chosun University, Gwangju, Republic of Korea

**Keywords:** *Geranium thunbergii*, cell cycle arrest, apoptosis, gastric cancer

## Abstract

*Geranium thunbergii* is a traditional East Asian medicine for stomach diseases including dysentery and stomach ulcers in East Asia and has been reported to possess biological activity. The benefits of *G. thunbergii* in gastric cancer are unknown. In this study, we demonstrate that *G. thunbergii* extract suppresses proliferation and induces death and G1/S cell cycle arrest of gastric cancer cells. Proliferation was significantly inhibited in a time- and dose-dependent manner. Cell cycle arrest was associated with significant decreases in CDK4/cyclinD1 complex and CDK2/cyclinE complex genes expression. In addition, the protein expression of caspase-3 was decreased and that of activated poly (ADP-ribose) polymerase (PARP) was increased, which indicated apoptosis. The expressions of the Bax and Bcl-2, which are apoptosis related proteins, were upregulated and down-regulated, respectively. The results indicate that *G. thunbergii* extract can inhibit proliferation and induce both G/S cell cycle arrest and apoptosis of gastric cancer cells. Also, the induction of apoptosis involved the intrinsic pathways of the cells. Take the results, we suggest that *G. thunbergii* extract has anti-gastric cancer activity and may be a potential therapeutic candidate for gastric cancer.

## Introduction

Gastric cancer is a common malignant tumor worldwide (Sitarz et al. 2018; Russo and Strong 2019). Despite advancements in treatment options, including chemotherapy, surgical treatment, and adjuvant therapies, the 5-year survival rate for gastric cancer patients remains relatively low (Izuishi and Mori 2016). Drug treatments and surgery still have limited benefit (Gao et al. 2016; Izuishi and Mori 2016). Therefore, improved treatment of gastric cancer is necessary. The use of bioactive agents in novel potential treatments is worth exploring.

Phytochemicals and natural products present in a wide range of some fruits and vegetables that are consumed daily have inhibitory effects on various cancers at the molecular and cellular levels (Mukherjee et al. 2001; Pal et al. 2012; Wang et al. 2012). *Geranium thunbergii* is a medicinal herb that has been traditionally used as a remedy for intestinal disorders in East Asia. *G. thunbergii* has also been reported to have various biological activities (Liu et al. 2006; Sung et al. 2011). There are two ways to use *G. thunbergii* medicinally. The dried aerial parts can be used to make a tea, or the dried plant can be boiled in water (Hiramatsu et al. 2004). The tea and boiled dried plant preparations are used to treat constipation and diarrhea, respectively, and also to prevent gastritis (Liu et al. 2006). The ability of *G. thunbergii* to suppress cancer cell growth is primarily mediated through the induction of apoptosis in lung adenocarcinoma (Li et al. 2013). As such, *G. thunbergii* is generally used as a therapeutic agent for digestive system diseases and has an anti-cancer mechanism, but interestingly, there is no research on its relationship with gastric cancer and the mechanism its effect on gastric cancer. Therefore, we focused on role of *G. thunbergii* in gastric cancer.

The failure to control cancer cell death associated with the induction of apoptosis and cell cycle arrest is considered the main limitation of cancer therapy (Evan and Vousden 2001; Nawab et al. 2012; Ehrhardt et al. 2013; Jung et al. 2018). Apoptosis is a type of programed cell death and is a physiological homeostatic mechanism (Konopleva et al. 1999; Green 2017). As a result of apoptosis, unwanted cells are eliminated in a well-organized sequential process (Konopleva et al. 1999; Green 2017). Caspases are central components of the apoptotic machinery in the proteolytic system (Konopleva et al. 1999). Apoptosis induces the activation of caspase-3 that subsequently cleaves its substrates, including poly-(ADP-ribose) polymerase (PARP), ultimately leading to apoptosis (Los et al. 2002). The cell cycle progresses in several stages—the G1, S, G2, and M phases—and is regulated by the activation of complexes involving cell cycle proteins (cyclins) and cyclin-dependent kinases (CDKs) (Nakanishi 2001 Barnum and O’Connell 2014). Since uncontrolled CDKs are often the cause of cancer, their function is tightly regulated by cell cycle inhibitors, such as p21^CIP/WAF^ and p27^KIP1^ proteins (Barnum and O’Connell 2014). Therefore, cell cycle arrest can be triggered by various stimulating factors, and may result in the blockage of cell division, cell death, and/or apoptosis

In this study, we confirmed the effect of *G. thunbergii* on *in vitro* anti-cancer activity using gastric cancer cell lines. We also investigated the molecular mechanism that underlies *G. thunbergii* extract-induced apoptosis and G1 cell cycle arrest against YCC-2 and SNU668 gastric cancer cells. The results indicate the value of *G. thunbergii* extract for the prevention of gastric cancer cell growth.

## Materials and methods

### Preparation of G. thunbergii methanol extract

Dried *G. thunbergii* was purchased from Cheongmyeong Yakcho Yeoju (Korea). It was extracted with 80% (v/v) methanol at 69°C for 3 h. This crude extract was dissolved in dimethyl sulfoxide.

### Cell culture

Six human gastric cancer cell lines (AGS, MKN-28, YCC-2, SNU-216, SNU-601, and SNU-668) were obtained from the Korea Cell Line Bank. All cells were cultured in RPMI-1640 medium (Welgene, Korea) containing 5% fetal bovine serum (Corning Costar, USA) and 1% antibiotic-antimycotic (Gibco, USA) in a 37°C incubator in an atmosphere of 5% CO_2._

### Cell proliferation assay

Cell proliferation after treatment with *G. thunbergii* extreact was determined using the WST-1 assay. Six human gastric cancer cells were seeded in wells of 96-well plates (1 × 10^4^ cells/well). After 24 h of incubation, cells were treated with *G. thunbergii* extract (0, 50, 100, 200, 300, 400, and 500 μg/mL) for 24, 48, and 72 h. WST-1 solution (EZ-cytox; Daeil, Korea) was added to each well and incubated at 37°C for 2 h. The absorbance was measured in an ultraviolet spectrophotometer at 450 nm.

### Crystal violet staining

YCC-2 and SNU-668 cells were seeded in 6-well culture plates (2 × 10^5^ cells/well). After 24 h of incubation, *G. thunbergii* extract (250 μg/mL) was added and the plates were incubated at 37°C for 48 h. The cells were washed with 1 × Phosphate buffered saline (PBS) and fixed in 1% glutaraldehyde (Sigma-Aldrich) for 10 min at room temperature (RT). After fixation, cells were washed with 1 ×  PBS and stained with 0.5% crystal violet (Sigma-Aldrich) for 10 min at RT.

### Cell cycle analysis

YCC-2 and SNU-668 cells were seeded in culture plates and incubated for 24 h at 37°C. The cells were then treated with *G. thunbergii* extract (250 μg/mL) for 24 and 48 h. After incubation, the cells were washed with 1 × PBS and fixed in 5 mL of 75% ethanol overnight at −20°C. After fixation, the cells were washed twice with cold 1 × PBS and dispersed in a staining solution containing 50 μg/mL propidium iodide (PI) and 50 μg/mL RNase A in PBS for 15 min at RT. Cell cycle distribution was measured by PI staining using a CytoFLEX flow cytometer (Beckman Coulter, USA).

### Apoptosis assay

YCC-2 and SNU-668 cells were plated in culture plates and treated with *G. thunbergii* extract (250 μg/mL) at 37°C for 48 h. The cells were harvested and washed twice with cold 1 × PBS. An apoptosis detection assay was performed using a fluorescein isothiocyanate (FITC)-Annexin V Apoptosis Detection Kit (BD Bioscience). Cells were resuspended using 1 mL of 1 × Binding buffer containing 5 μL of Annexin V and PI solution and incubated for 20 min at RT in the dark. Apoptosis distribution after *G. thunbergii* treatment was measured by Annexin V staining using the aforementioned CytoFLEX flow cytometer.

### Western blotting

YCC-2 and SNU-668 cells were treated with *G. thunbergii* extract (250 μg/mL) for 48 h. RIPA buffer was added to extraction protein from cells. After incubation at 4°C for 35 min, each sample was centrifuged at 13,000 rpm and 4°C for 25 min, and the protein concentration was measured by the bovine serum albumin (BSA) Protein Assay (Thermo Fisher Scientific, USA). A defined quantity of total protein was subjected to 10% or 12% SDS-PAGE, and the resolved protein bands were transferred to polyvinylidene fluoride membranes. Each membrane was blocked using 5% skim milk in phosphate buffered saline containing 0.1% Tween-20 (PBST) for 2 h. Each membrane was incubated with primary antibody diluted 1:1000 in 5% BSA in PBS containing Tween-20 buffer overnight at 4°C. This was followed by exposure of each membrane to secondary antibody (1:5000 dilution in PBST) for 1.5 h at RT followed by enhanced chemiluminescence to detect binding of both antibodies (Bio-Rad, Korea).

### Antibodies

Monoclonal antibodies against Bcl-2 and Bax were purchased from Santa Cruz Biotechnology (USA). The polyclonal antibodies used were against CDK4 (Santa Cruz Biotechnology), cyclin D1 (Santa Cruz Biotechnology), CDK2 (Santa Cruz Biotechnology), cyclin E (Santa Cruz Biotechnology) caspase-3 (GeneTex, USA), poly ADP-ribose polymerase (PARP, GeneTex), and glyceraldehyde-3-phosphate dehydrogenase (GAPDH, Bioworld Technology, USA).

### Reverse transcription-polymerase chain reaction (RT–PCR) analysis

Total RNA was extracted from YCC-2 and SNU-668 cells treated with *G. thunbergii* using RNAiso Plus reagent (TaKaRa Bio, Japan), and cDNA was synthesized using cDNA Master Mix (ToYoBo, Japan) according to the manufacturer’s protocol. Reverse transcription PCR was performed as described previously (Kim et al. 2018) using the following PCR primer sequences: Bcl-2, 5ʹ-GGATGCCTTTGTGGAAAACCCTGT-3ʹ (forward) and 5ʹ-AGCCTGCAGCTTTGTTTCAT-3ʹ (reverse); Bax, 5ʹ-TTTGCTTCAGGGTTTCATCC-3ʹ (forward) and 5ʹ-CAGTTGAAGTTGCCGTCAGA-3ʹ (reverse); caspase-3, 5ʹ-TTTTTCAGAGGGGATCGTTG-3ʹ (forward) and 5ʹ-CGGCCTCCACTGGTATTTTA-3ʹ (reverse); PARP, 5ʹ-TGGAACATCAAGGACGAGCT-3ʹ (forward) and 5ʹ-GCATCGCTCTTGAAGACCAG-3ʹ (reverse); GAPDH, 5ʹ-GGCTGCTTTTAACTCTGGTA-3ʹ (forward) and 5ʹ-ACTTGATTTTGGAGGGATCT-3ʹ (reverse). PCR products were used for 1% agarose gel electrophoresis with RedSafe Nucleic Acid Staining Solution (iNtRON Biotechnology).

### Statistical analysis

Analysis of data was performed using GraphPad Prism5 software (GraphPad, USA). Differences between treated and control samples were analyzed using the Student’s t-test. A *P*-value less than 0.05 was considered statistically significant.

## Results

### G. Thunbergii extract suppresses proliferation of gastric cancer cells

**Figure 1.**
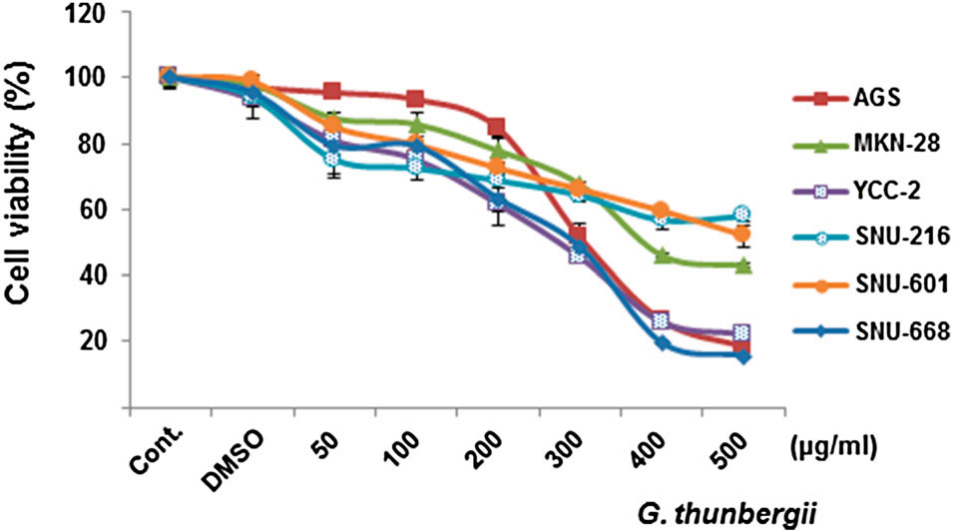
***G. thunbergii* extract inhibits gastric cancer cell proliferation in a dose-dependent manner.** WST-1 assays were performed to detect cell viability in the six gastric cancer cell lines treated for 48 h with *G. thunbergii* extract (50, 100, 200, 300, 400, and 500 μg/mL).

A preliminary screening using the WST-1 assay was done to determine the effect of *G.thunbergii* extract on proliferation of six gastric cancer cell lines (AGS, YCC-2, MKN-28, SNU216, SNU601, and SNU-668). Treatment with 50, 100, 200, 300, 400, and 500 μg/mL of the extract for 48 h significantly decreased the proliferation of all gastric cancer cells in a dose-dependent manner (Figure 1). The IC_50_ values are summarized in Table 1. In subsequent experiments, YCC-2 and SNU-668 cells were used since they were most affected by *G. thunbergii* extract. Furthermore, 250 μg/mL concentration of *G. thunbergii* extract was used.

**Table 1.** IC_50_ values for G. thunbergii extract **in WST-1 assays with six gastric cancer cell lines treated for 48 h.**

Cell line	IC_50_ (95% CI)	*R*^2^
AGS	308.8 (301.0–316.8) ug/ml	0.9846
MKN-28	423.1 (390.4–458.4) ug/ml	0.9108
YCC-2	228.3 (211.6–246.3) ug/ml	0.9322
SNU-216	1146 (836.4–157.1) ug/ml	0.8069
SNU-601	647.5 (588.5–712.4) ug/ml	0.9535
SNU-668	231.7 (207.3–258.9) ug/ml	0.8574

Notes: Data are mean ± SD values for five independent experiments; IC_50_ values of human gastric cancer cell lines; 95% CI = 95% Confidence Intervals.

### G. Thunbergii extract reduces cell proliferation in a time-dependent manner

**Figure 2.**
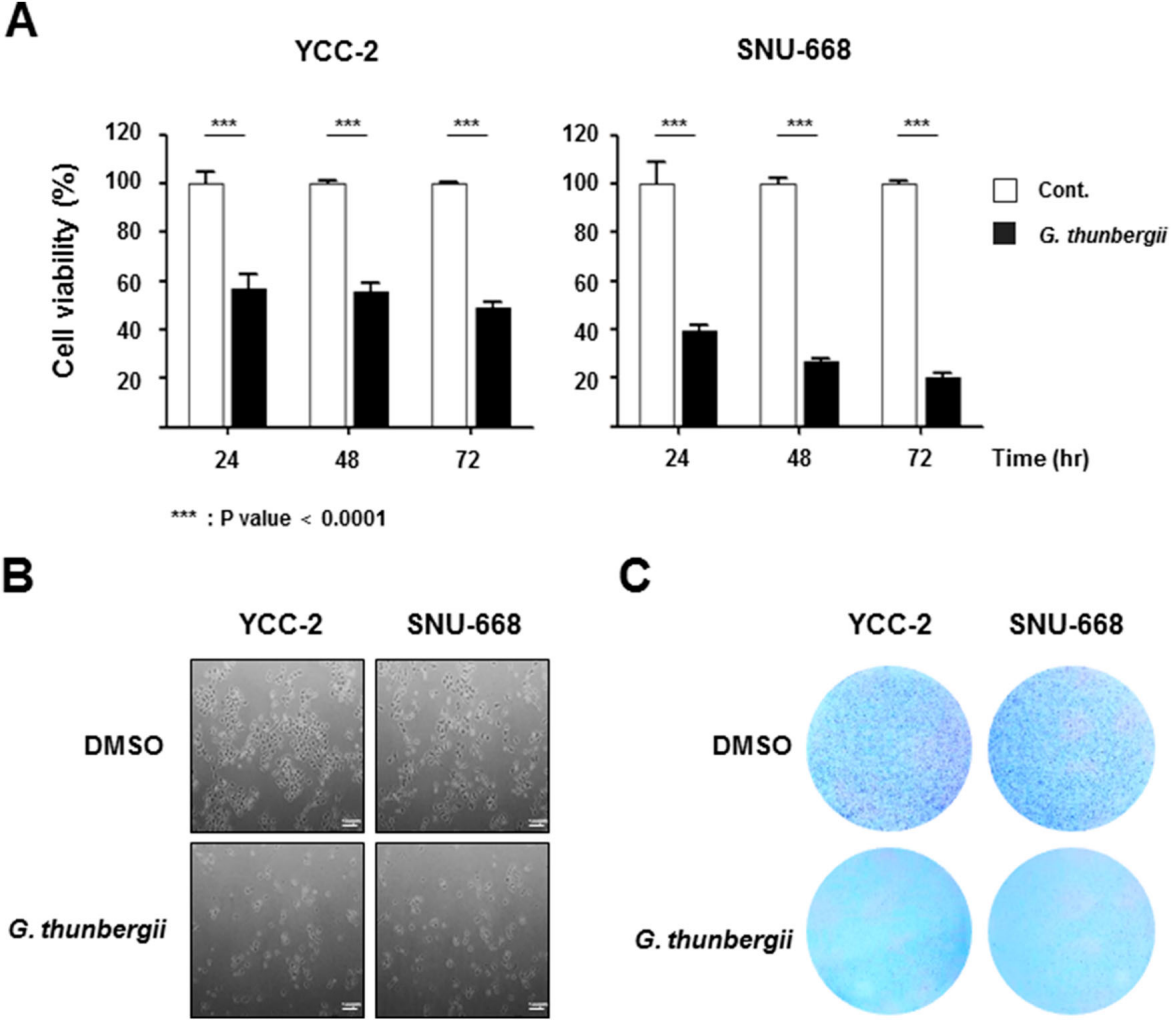
***G. thunbergii* extract reduces gastric cell proliferation in a time-dependent manner.** (A) WST-1 assays were performed to detect the viability of YCC-2 and SNU-668 cells treated with *G. thunbergii* extract (***P < 0.0001). (B) and (C) After treatment with *G. thunbergii* extract treatment for 48 h, the morphology and density of YCC-2 and SNU-668 cells were assessed by microscopy and crystal violet staining. Scale bar represents 100 μm.

The WST-1 assay was also used to determine the temporal effects of *G. thunbergii* extract on the proliferation of YCC-2 and SNU-668 cells. The results revealed that *G. thunbergii* extract markedly inhibited the proliferation of both cell types, with significant differences between the treatment groups and the control group evident at each time point (all *P* < 0.01) (Figure 2A). The findings were corroborated by microscopy examination of samples of *G. thunbergii* extract treated cells (Figure 2B). Crystal violet experiments also revealed decreased gastric cancer cell growth in the presence of extract (Figure 2C).

### G. Thunbergii extract induces cell cycle arrest at G1/S phase in gastric cancer cells

**Figure 3.**
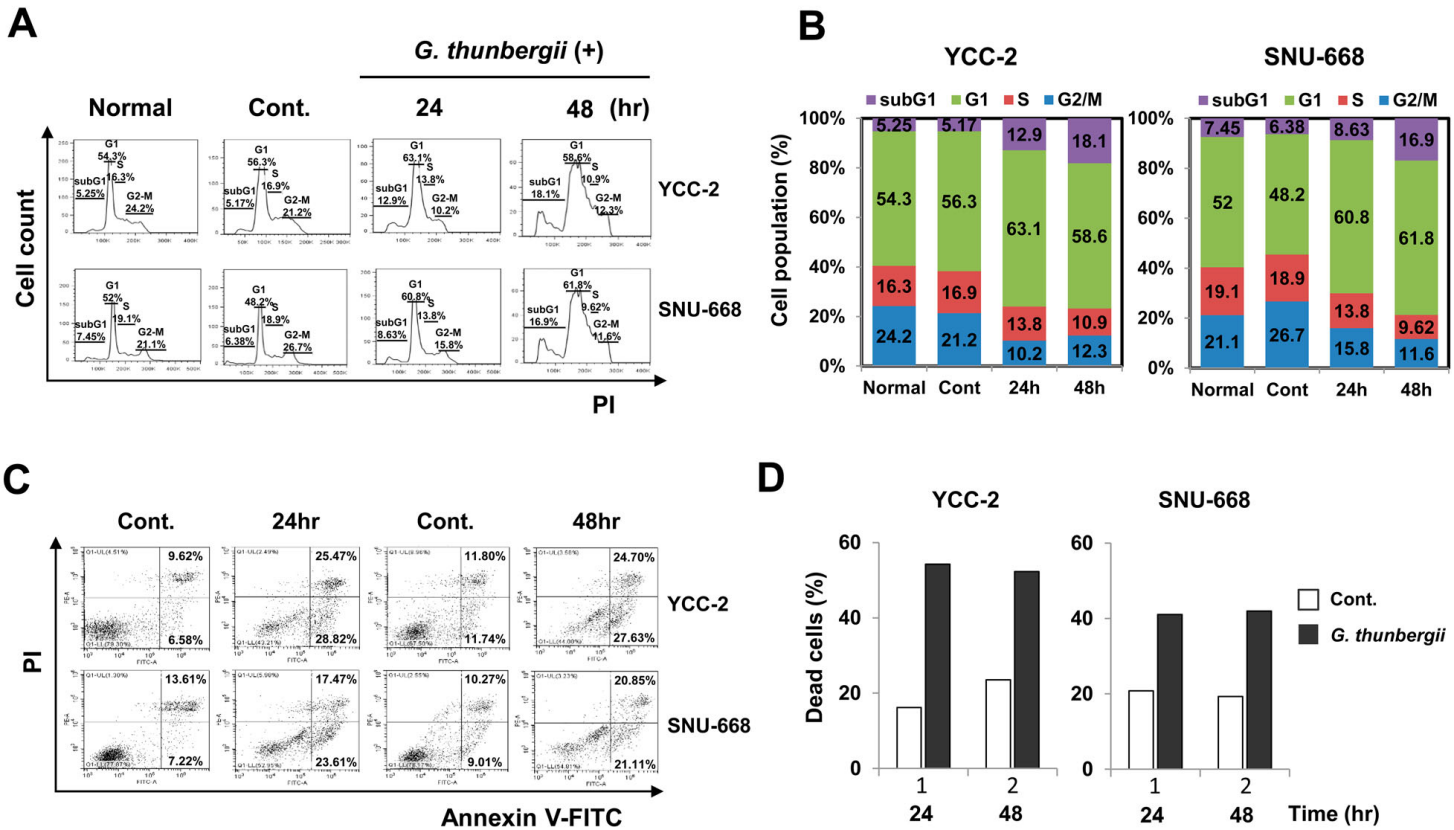
***G. thunbergii* extract arrests the cell cycle and induces apoptosis in gastric cancer cells.** (A) Apoptosis rates of YCC-2 and SNU-668 cells were detected by fluorescence-activated cell sorting analysis using Annexin V-FITC/PI staining. (B) Quantitative data of cell cycle apoptosis. (C) YCC-2 and SNU-668 cell cycles detected using PI staining. (D) Quantitative cell cycle data.

Cell proliferation is correlated with the regulation of cell cycle progression (Foster et al. 2010). Through the Figure 1 result concerning the growth inhibition effect of gastric cancer cells by *G. thunbergii* extract prompted the assessment of whether this growth inhibition was triggered by cell cycle arrest. To explore this, we analyzed the cell cycle distribution by flow cytometry with PI staining. Cells harvested at 24 or 48 h were analyzed (Figure 3A and B). The cycle phase ratio was also determined. YCC-2 and SNU-668 cells treated with *G. thunbergii* extract displayed an increased percentage of the cell population in the subG1 and G1 phases and a decreased percentage of cells in the S phase (Figure 3A and B). The proportion of cells in the subG1 and G1 phase increased to nearly 15% in both YCC-2 and SNU-668 cell populations in the presence of 250 μg/mL *G. thunbergii* extract compared with the control (Figure 3A and B). The results suggested that *G. thunbergii* extract effectively induced cell cycle arrest in the two gastric cancer cell types.

### Effect of G. thunbergii extract on G1 phase related protein expression

**Figure 4.**
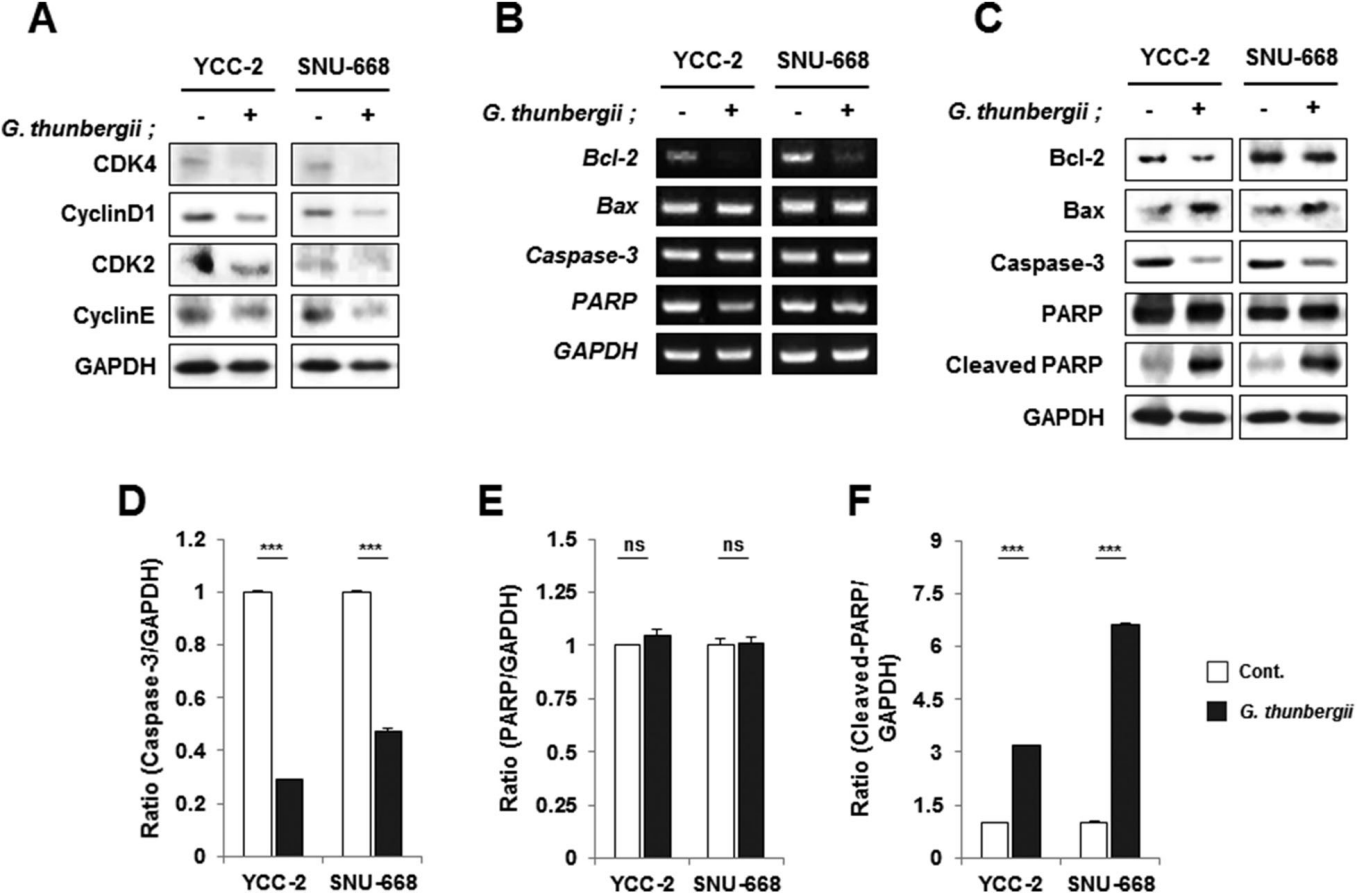
Gene and protein expression associated with apoptosis- and G1/S arrest in gastric cancer cells following treatment with *G. thunbergii* extract. (A) Proteins related to apoptosis and G1 arrest were detected by western blot. YCC-2 and SNU-668 cells were treated with *G. thunbergii* extract for the indicated times. (B) RT-PCR detection of apoptosis related genes after 48 h treatment with *G. thunbergii* extract. (C) Detection of apoptosis related genes after 48 h treatment with *G. thunbergii* extract using by western blot. (D-F) Relative protein expression levels of (E) Caspase-3, (E) PARP, and (F) cleaved PARP in gastric cancer cells following treatment with *G. thunbergii* extract (***P < 0.0001, *P < 0.05, ns: not significant).

We next investigated whether the cell cycle arrest at G1 phase produced by the *G. thunbergii* extract was related to the expression of cell cycle regulatory genes, such as CDK4/cyclinD1 complex and CDK2/cyclinE complex, which are essential for cell cycle progression from the G1 phase to the S phase. Western blot data suggested that *G. thunbergii* extract reduced the protein levels of CDK4, CDK2, cyclinD1 and cyclinE (Figure 4A). The data indicated a correlation of the cell cycle arrest induced by *G. thunbergii* extract with its inhibition of G1 phase related genes and proteins in gastric cancer cells.

### G. Thunbergii extract induces apoptosis in gastric cancer cells

To elucidate whether the effects of *G. thunbergii* extract on the proliferation and viability of gastric cancer cells were associated with induction of apoptosis, the prevalence of apoptotic cells was determined using Annexin V staining, following treatment of YCC-2 and SNU-668 cells with 250 μg/mL of *G. thunbergii* extract. Gastric cancer cells treated with *G. thunbergii* extract for 24 and 48 h were examined using microscopy (Figure 3C). The percentage of apoptotic cells increased to over 25% and 20% in the YCC-2 and SNU-668 populations, respectively (Figure 3D). Similarly, the number of apoptotic cells appeared to show a time-dependent increase in response to *G. thunbergii* extract treatment. Thus, the anti-proliferative effect of *G. thunbergii* extract on gastric cancer cells could be associated with the induction of apoptosis.

### Effect of G. thunbergii extract on Bax and Bcl-2 expression

To determine if Bax and Bcl-2 participated in the regulation of apoptosis initiation (Autret and Martin 2009), western blot analysis was performed to determine the levels of the Bax and Bcl-2 proteins in cells treated for 48 h with *G. thunbergii* extract. The expression of Bax protein was increased, and the expression of Bcl-2 protein was decreased; both changes were statistically significant compared to the control group (*P* < 0.01; Figure 4B and C). The results suggested that *G. thunbergii* extract could decrease Bcl-2 expression and increase Bax expression to regulate or induce gastric cancer cell apoptosis.

### G. Thunbergii extract induces activation of caspases and PARP

Caspase family members, including caspase 3, as well as downstream substrates, such as PARP, are crucial mediators of the apoptotic process (Konopleva et al. 1999). Given our demonstration of the significant decrease in gastric cancer cell viability after 48-h exposure to *G. thunbergii* extract, we selected this time to determine whether induction of apoptosis in gastric cancer cells treated with *G. thunbergii* extract was associated with the activation of caspases and PARP using western blotting. The expression of procaspase 3 decreased and PARP underwent cleavage, thereby confirming apoptosis (Figure 4C and D). Although pro-PARP has not changed (Figure 4C and E), however, cleaved-PARP expression of was increased by *G. thunbergii* extract (250 μg/mL) treatment (Figure 4C and F). These data suggest that apoptosis induced by *G. thunbergii* extract in gastric cancer cells is associated with caspase 3 activation.

## Discussion

The relationship between natural products and anti-cancer drugs is being scrutinized in various cancers, including gastric cancer (Mukherjee et al. 2001; Khan 2014; Ijaz et al. 2018). *G. thunbergii* a naturally occurring plant in Northeast Asia, especially South Korea. Plant extract is commonly used for the relief of stomach ailments, including dysentery and stomach ulcers (Liu et al. 2006). However, whether *G. thunbergii* extract has any benefit in the treatment of gastric cancer is unknown. The aim of this study was to explore the mechanism underlying the *G. thunbergii* extract-induced death of human gastric cancer cells. Here, we report that treatment of gastric cancer cells with *G. thunbergii* extract inhibits cell proliferation and viability, blocks the cell cycle, and induces apoptosis. In cancer, including gastric cancer, the formation of a tumor is the result of the loss of balance between cell proliferation and apoptosis. Thus, inhibiting cell proliferation and inducing apoptosis is an important approach in tumor prevention and treatment (Magi-Galluzzi et al. 1998; Jackson and Evers 2009).

In a preliminary study, *G. thunbergii* extract markedly reduced the proliferation of gastric cancer cells (Figure 1), raising the possibility that *G. thunbergii* extract might be a potential therapeutic agent. Several naturally occurring phytochemicals have been reported to suppress the growth of cancer cells by disrupting cell cycle progression and promoting apoptosis. Cancer cell proliferation is mainly regulated by the cell cycle (Evan and Vousden 2001), which comprises four distinct sequential phases (subG1, G1, S and G2/M) (Gamet-Payrastre et al. 2000). Cell cycle progression is tightly controlled by an accurate serial activation of cyclins, CDKs, and cyclin-dependent kinase inhibitors. In tumor cells, cell cycle deregulation abrogates differentiation and produces abnormal cell growth. The transition from the G1 to S phase is responsible for the initiation and completion of DNA replication, which is regulated by activation of cyclin D1/CDK4 complex and cyclinE/CDK2 complex (Bertoli et al. 2013).

To further investigate the molecular basis by which *G. thunbergii* extract inhibited the G1 transition in tumor cells, we analyzed the expression of cyclins and CDKs involved in cell cycle regulation (Jackson and Evers 2009). *G. thunbergii* extract markedly reduced the expression levels of cyclin D1 and CDK4 proteins. These findings support the view that *G. thunbergii* extract inhibits the growth of gastric cancer cells by arresting cells at the G1 phase.

Apart from cell cycle arrest, induction of apoptosis is viewed as a mechanism through which a variety of naturally occurring phytochemicals inhibit tumor growth (Evan and Vousden 2001). We used PI staining to detect the induction of the subG1 population of gastric cancer cells treated with *G. thunbergii* extract (Figure 3A and B). The G1 phase was also induced. The findings confirmed the ability of *G. thunbergii* extract to induce apoptosis. The expression of the apoptosis related proteins PARP, caspase-3, Bax, and Bcl-2, was analyzed by western blotting (Zhao et al. 2006). In gastric cells treated with *G. thunbergii* extract, the expression of the apoptosis-promoting protein Bax was upregulated, that of the apoptosis-inhibiting protein Bcl-2 was down-regulated, and that of the apoptosis effector protein caspase-3 was greatly activated by the activated PARP protein. These changes triggered the apoptotic process of programed cell death. These results suggested that *G. thunbergii* extract induces gastric cell apoptosis through the activation of caspase-3 and that the initiation of apoptosis is regulated by PARP, Bax, and Bcl-2 proteins (Zhao et al. 2006). In summary, *G. thunbergii* extract inhibits the proliferation of gastric cancer cells by inducing G1 phase cell cycle arrest and by activating apoptosis. Especially, the down-regulated expression of cyclin D1 and CDK4 supports the mechanism of G1 phase cell cycle arrest. The decreases of total caspase-3 and Bcl-2 and the increases of cleaved PARP and Bax activate apoptosis. The collective findings suggest that *G. thunbergii* extract has anti-gastric cancer activity and may be a potential therapeutic candidate for gastric cancer.
